# Impact of serotonin transporter (SERT) binding affinity on the risk of libido disorders related to antidepressants

**DOI:** 10.1192/j.eurpsy.2022.716

**Published:** 2022-09-01

**Authors:** R. Zeiss, M. Gahr, H. Graf

**Affiliations:** University of Ulm, Department Of Psychiatry, Ulm, Germany

**Keywords:** sexual disorder, libido disorder, adverse drug reaction, pharmacovigilance

## Abstract

**Introduction:**

Sexual dysfunction is a frequent adverse drug reaction (ADR) of antidepressants that considerably affects quality of life and adherence to therapy. We previously investigated the potential underlying neurofunctional mechanisms by neuroimaging methods and revealed a dampening of the dopaminergic mesolimbic-mesocortical reward system along with decreased sexual functioning under serotonergic antidepressants compared to placebo.

**Objectives:**

Within a combined pharmacoepidemiologic and pharmacodynamic approach, we examined the association between serotonin transporter (SERT) affinity of various antidepressants and corresponding alterations in sexual desire as ADR.

**Methods:**

Using disproportionality analyses, reporting odds ratios (RORs) were calculated for reports indicating decreased sexual desire as ADR under the antidepressants. The data were extracted from the WHO global database of individual case safety reports VigiBase and several MedDRA terms were grouped for “Sexual Desire Disorders”. For the pharmacodynamic assessment, we calculated Pearson correlation coefficients between SERT affinity and corresponding RORs

**Results:**

16 signals were detected for “Sexual Desire Disorders”. We observed a statistically significant (r (20) =. 65, p = 0.001) association between SERT affinity and decreased sexual desire. Higher SERT affinity was associated with higher risk of sexual desire.

**Conclusions:**

While sexual dysfunctions under serotonergic medication were previously described, we now elaborated that in particular attenuated sexual desire as ADR is associated with SERT affinity of the antidepressant. These results strengthen our previously described neurofunctional model regarding sexual dysfunction under antidepressant medication and indicate that the specific SERT affinity of the antidepressant drug should be considered in clinical practice to minimize the risk of this ADR.

**Disclosure:**

No significant relationships.

